# Vascular Cast to Program Antistenotic Hemodynamics and Remodeling of Vein Graft

**DOI:** 10.1002/advs.202204993

**Published:** 2023-02-02

**Authors:** Hyunsu Ha, Ju Young Park, Chan Hee Lee, Deok‐Hyeon Son, Soon Won Chung, Sewoom Baek, Kyubae Lee, Kang Suk Lee, Se Won Yi, Mi‐Lan Kang, Dae‐Hyun Kim, Hak‐Joon Sung

**Affiliations:** ^1^ Department of Medical Engineering Yonsei University College of Medicine 50–1 Yonsei‐ro Seodaemun‐gu Seoul 03722 Republic of Korea; ^2^ TMD LAB Co. Ltd. 6th floor, 31, Gwangnaru‐ro 8‐gil, Seongdong‐gu Seoul 03722 Republic of Korea; ^3^ Department of Plastic Surgery Korea University Guro Hospital Korea University College of Medicine Gurodong‐ro 28‐gil Guro‐gu Seoul 08308 Republic of Korea; ^4^ Department of Brain Korea 21 FOUR Project for Medical Science and Medical Engineering Yonsei University College of Medicine 50–1 Yonsei‐ro Seodaemun‐gu Seoul 03722 Republic of Korea; ^5^ Department of Veterinary Surgery Chungnam National University College of Veterinary Medicine 99, Daehak‐ro Yuseong‐gu Daejeon 34134 Republic of Korea

**Keywords:** antistenosis, artery‐vein graft, bridge structure, computational simulation, elastic fixity, vascular wall device

## Abstract

The structural stability of medical devices is established by managing stress distribution in response to organ movement. Veins abruptly dilate upon arterial grafting due to the mismatched tissue property, resulting in flow disturbances and consequently stenosis. Vascular cast is designed to wrap the vein‐artery grafts, thereby adjusting the diameter and property mismatches by relying on the elastic fixity. Here, a small bridge connection in the cast structure serves as an essential element to prevent stress concentrations due to the improved elastic fixity. Consequently, the vein dilation is efficiently suppressed, healthy (laminar and helical) flow is induced effectively, and the heathy functions of vein grafting are promoted, as indicated by the flow directional alignment of endothelial cells with arterialization, muscle expansion, and improved contractility. Finally, collaborative effects of the bridge drastically suppress stenosis with patency improvement. As a key technical point, the advantages of the bridge addition are validated via the computational modeling of fluid–structure interaction, followed by a customized ex vivo set‐up and analyses. The calculated effects are verified using a series of cell, rat, and canine models towards translation. The bridge acted like “Little Dutch boy” who saved the big mass using one finger by supporting the cast function.

## Graphical abstract

When veins and arteries are grafted in patients undergoing hemodialysis or vascular disease, the veins abruptly dilate abruptly owing to the weak mechanical properties to handle arterial flow and pressure. Vein dilation causes blood flow disturbance and vessel occlusion. The vascular cast externally wraps the arteriovenous graft by covering the connection part to manage the dilation changes and prevents vascular occlusion.

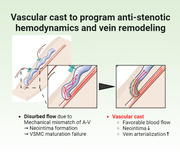

## 1 Introduction

An abnormal stress concentration can lead to gradual structural collapse over time if left unaddressed. The concentration can be relieved when the stress is efficiently distributed over the entire structure.^[^ ^1^ ^]^ Medical devices are implanted into the body in places subjected to dynamic movements with continuous stress delivery on the devices. Managing the propagated stress is critical for stabilizing the device structure, and allowing it to operate effectively during the implantation period. Particularly, when a vein is grafted to an artery, the vigorous pulsatile flow and pressure from the artery side induce vein dilation due to the mismatched mechanical properties.^[^ ^2^ ^]^ The vein dilation generates abnormal circumferential pressure with a sudden change of inner diameter throughout the anastomosis part from the vein to the artery. This process inevitably leads to flow disturbances and consequently stenosis with aggressive intimal hyperplasia.^[^ ^3^ ^]^ Hence, the suppression of vein dilation by managing the circumferential tension is suggested as a solution to prevent this pathological process.

Moreover, preservation of arterial flow through the vein part presents another challenge for maintaining the long‐term patency of grafting.^[^ ^4^ ^]^ As a considerable solution, the vein can undergo arterialization in endothelial cells (ECs) and wall remodeling. First, the flow directional alignment of ECs indicates the formation of healthy laminar flow with efficient handling of EC permeability to prevent vascular lipid deposition.^[^ ^5^ ^]^ Venous ECs can express phenotypic markers of arterial ECs upon exposure to arterial flow due to the cell plasticity for adapting to the hemodynamic environment, which is specific to vessel type, size, and position.^[^ ^6^ ^]^ Second, the venous wall can be arterialized to expand the media muscle towards the adventitia side, and thereby improve the contractile synchronization with the arterial flow.^[^ ^7^ ^]^ The successful vein grafting requires maintenance of the contractile phenotype of smooth muscle cells (SMCs), guidance of SMC migration towards the outer wall instead of the intima side, and regeneration of the microvasculature (vasa vasorum) on the outer ‐wall to prevent the hypoxic changes in the SMC phenotype.^[^ ^8^ ^]^ These orchestrated processes indicate a need to place the controller onto the outer‐ wall, thereby enabling an effective and efficient outside‐in control.

To prevent stenotic remodeling, the vascular cast was designed to preserve arterial flow profiles throughout the artery‐vein graft with consequent arterialization of the vein by wrapping the anastomosis site, representing the unprecedented function. As a key regulator of this function, the elastic fixity was derived from the unique cooperation of elastic shape memory polymer (SMP)^[^ ^9^ ^]^ with the structural fixing enforcement by the cast design. The vessel diameter expansion with consequent changes of flow pressure and the long‐curved structure like an aortic arch induces a flow disturbance. Hence, the fixity was required through shape programing with the structural fixing enforcement to suppress excessive vein dilation and flow disturbances by fixing the calculated structure and angle of vein grafting to an artery.^[^ ^10^ ^]^ Our strategy was to mimic a short version of the aortic arch to manage the vigorous arterial hemodynamics and minimize flow disturbances. As our thermo‐responsive SMP becomes more ductile after the shape recovery under the body temperature, the inter‐body elasticity of SMP enables continuous contractile synchronization with the pulsatile hemodynamics, and thereby preserves arterial flow parameters. The preservation of arterial flow profiles throughout the graft by these critical functions induced arterialization of the vein because most cells and tissues have the ability to adapt to new environments, such as transitioning from venous to arterial hemodynamics. The key design of vascular cast provides new knowledge to the field of cardiovascular medicine by enabling the outside‐in control of intimal hyperplasia from the vascular outer wall, significantly advancing current vascular devices.

A critical design point was strategized to operate the successful functions of vascular cast by relieving the stress concentrations to maximize the elastic fixity. Our proposed strategy is to add a bridge into the cast design in a strand form, which is set to act like one finger that saved dam collapsing (“Little Dutch boy”). As consequent advantages, (i) the improved elastic fixity played a pivotal role in suppressing the flow disturbance while preserving the healthy flow characteristics including pulsatile laminar and helical patterns. (ii) Arterialization of the vein was induced as demonstrated by the previously mentioned aspects. An interdisciplinary approach was applied to the present study as the whole set of bridge design‐function relationship is precalculated through computational modeling of the structure and hemodynamics together. The results were confirmed using a series of customized ex vivo, cell, rat, and canine models. This study suggests the paradigm‐shiftable development of implantable medical devices that can efficiently manage stress from the organ movement, thereby improving translational potential.

## 2 Results

The vascular cast was designed to wrap the artery‐vein grafts by covering the anastomosis part to prevent stenosis due to the improved elastic fixity of SMP through the bridge additions. The structure–function relationship between the cast bridge structure and hemodynamic function was simulated by their cocomputational modeling using the experimental results of input properties. The input parameters from the mechanical analyses of materials and vessels were processed through geometry construction using 3D CAD, meshing and discretization, and structural and CFD analyses (Figure S1a, Supporting Information). The elastic properties of SMP film were analyzed using dynamic mechanical analyzer (DMA) with a bilinear isotropic model as a reference, and the elastic parameter values were entered into the computational modeling analyses (Figure S1b, Supporting Information). After harvesting the inferior vena cava (IVC) and aorta from rabbits, the *ex vivo* system was set to calculate the vascular wall stress upon increasing the flow pressure using Laplace's law as a model for vein dilation (Figure S1c, Supporting Information).^[^ ^11^ ^]^ As a result, the hyperelastic parameter values of the vascular properties were analyzed using the Mooney–Rivlin model as a reference and were entered into the computer modeling analysis.^[^ ^12^ ^]^

**Figure 1:**
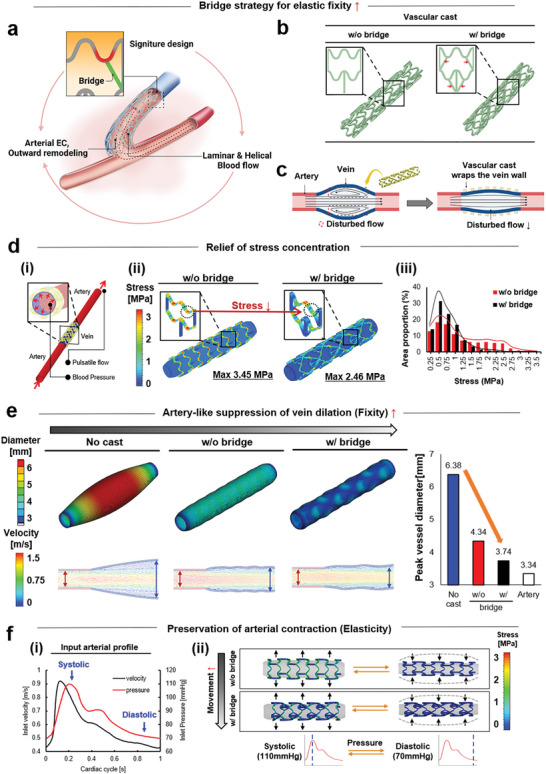
Computational modelling validation of bridge advantages in vascular cast deployment. a) The vascular cast is designed to wrap artery‐vein grafts by covering the anastomosis part to prevent stenosis by operating the elastic fixity function of shape memory polymer (SMP). As a signature design of the cast, a bridge is added to induce healthy (laminar and helical) flow and graft functions. b) These beneficial effects were exerted by placing the bridge in the indicated positions (red arrows). c) As a fundamental effect, the cast deployment suppresses vein dilation in response to vigorous arterial flow and pressure, thereby reducing flow disturbance. d) When a vein segment is grafted into an artery, (i) the vascular cast is designed to handle vein dilation‐derived circumferential pressure in response to the arterial flow and pressure. (ii) The computational analysis demonstrates that the bridge facilitates stress distribution and thereby relieves the concentration, as indicated by the reduction of the maximum stress from 3.45 MPa (w/o bridge) to 2.46 MPa (w/ bridge). (iii) The bridge causes an overall stress distribution in the lower range contrast to the high range of the sporadic distribution observed in the w/o bridge group. The elastic fixity of vascular cast represents a unique SMP‐endowed property, providing significant advantages to simultaneously suppress excess vein dilation and synchronize arterial contractility. e) In this regard, the bridge most effectively contributes to the suppression of vein dilation and consequently flow disturbance among the test groups, as supported by the arterial level of peak vessel diameter (“fixity”). f) The bridge also preserves (i) arterial flow profiles (velocity and pressure)^[^ ^29^ ^]^ (ii) by enhancing the cast movement due to the relief of stress concentrations and the reduction of overall stress ranges throughout the cast structure (“elasticity”).

The bridge addition induced healthy (laminar and helical) flow and graft functions by serving as the design signature of vascular casts (**Figure** **1**a); and the bridge position was determined to exert these advantageous effects (Figure 1b). Compared to the cast without (w/o) bridges, this design supported the cast to suppress vein dilation more effectively in response to vigorous arterial flow and pressure, thereby reducing the flow disturbance (Figure 1c). In vein‐to‐artery grafts (Figure 1d), the key role of vascular cast was set to manage the vein dilation‐derived circumferential pressure in response to the arterial flow and pressure. The computational simulation demonstrated that the bridge facilitated stress distribution and thereby relieved the stress concentration, as indicated by the reduction of maximum stress from 3.45 MPa (w/o bridge) to 2.46 MPa (w/ bridge). The bridge addition resulted in the lower ranges of overall stress distribution in contrast to the sporadic distribution that resulted in the high ranges seen in the w/o bridge group. The elastic fixity of vascular cast was driven by the unique properties of SMP and provided significant advantages in suppressing excess vein dilation while supporting synchronization of the cast with the arterial contractility. Consequently, the bridge was most effective at suppressing vein dilation and flow disturbance in comparison to other test groups, as evidenced by the arterial level of peak vessel diameter (“fixity”) (Figure 1e). The bridges also preserved (i) the arterial flow profiles (velocity and pressure) (ii) by enhancing the cast movement due to relieving the stress concentrations and reducing the overall stress on the cast structure (“elasticity”) (Figure 1f).

**Figure 2:**
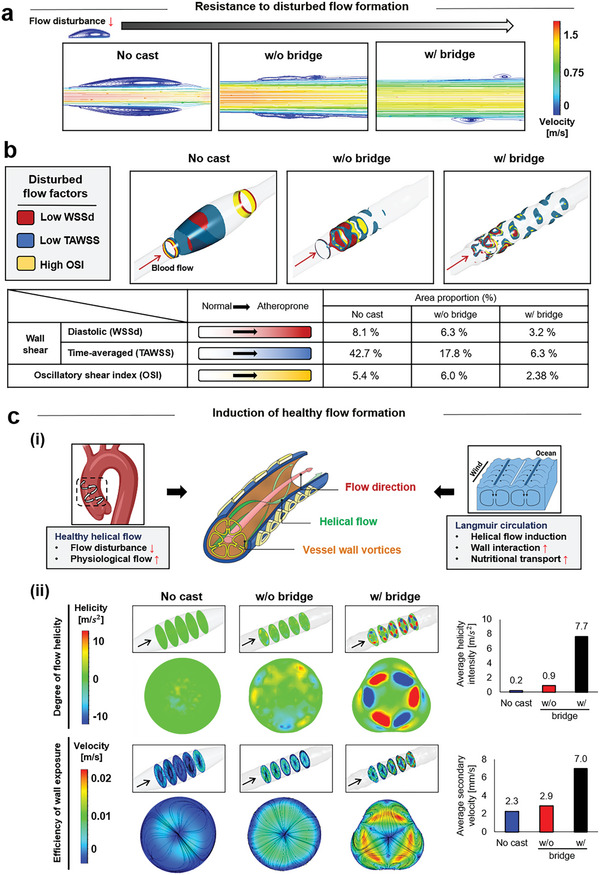
Computational fluid dynamics analysis of the bridge advantages in healthy flow formation. a) The bridge causes a drastic suppression in the flow disturbance at the wall side with a normal parabolic gradient of flow velocity compared to no cast and w/o bridge. b) The results are further confirmed by the most reduction in the diastolic and time‐averaged wall shear stress (WSSd and TAWSS) with the lowest oscillatory shear index (OSI), as observed in the w/ bridge group among the test groups. c) As healthy characteristics, (i) the physiological flow not only reduces the disturbance but also increases helicity, which is derived from the Langmuir circulation to increase the wall interaction and thereby to facilitate the nutrient transport. (ii) When the other groups were compared, the bridge significantly contributes to the increased formation of vortices around the wall and thereby prolong the interaction between the flow and the wall with a more efficient nutrient supply, as indicated by the helicity intensity (top) and secondary velocity (bottom).

The bridge addition significantly suppressed the flow disturbance on the wall side with a normal parabolic gradient of flow velocity compared to the no cast and w/o bridge group (**Figure** **2**a). When the hemodynamic descriptors were considered (Table S1, Supporting Information),^[^ ^13^ ^]^ the advantageous bridge effects were further evidenced by the most significant reduction in the diastolic and time‐averaged wall shear stress (WSSd and TAWSS) with the lowest oscillatory shear index (OSI) compared to the other test groups (Figure 2b).^[^ ^14^ ^]^ The healthy physiological flow not only reduced the disturbance, but also increased the helicity, which was derived from the Langmuir circulation to enhance the flow staying on the vascular wall with the facilitating of nutrients transport (Figure 2c; Figure S2, Supporting Information).^[^ ^15^ ^]^ Compared to the other groups, the bridge addition significantly increased the formation of vortices around the wall and prolonged the interaction between the flow and wall with a more efficient nutrient supply, as indicated by the helicity intensity and secondary velocity.

A library of poly (caprolactone‐*co*‐glycidyl methacrylate) was synthesized via the ring‐opening polymerization of CL with GMA by controlling the molar ratio of the components (6‐arm *x*% PCL – *y*% PGMA, % molar ratio) (Figure S3a, Supporting Information). As a new design, the SMP structure was produced to branch out in a form of six arms so that the arms promoted integrations among the polymer chains through crosslinking to improve the elastic fixity of vascular cast. The 6‐arm *x*% PCL – *y*% PGMA was successfully synthesized, as supported by the ^1^H‐NMR spectra according to the molar ratio in CDCl_3_; : *δ* = 6.13 [s, = CH_2_, (G2)], 5.58 [s, = CH_2_, (G1)], 4.10 [m, ‐OCH_2_, (A)], 2.41 [m, ‐CH_2_, (E)], 1.97 [s, ‐CH_3_, (F)], and 1.74 [m, ‐CH_2_, (B, D)], and 1.45 [m, ‐CH_2_] (Figure S3b, Supporting Information). Their thermal properties were tuned by changing the molar ratio and crosslinking, as characterized by DSC (Figure S3c, Supporting Information). The 1‐year degradation property of SMP was characterized under an accelerated aging condition following the American Society of Testing and Materials (ASTM) international standard 1980 (Figure S3d, Supporting Information). When the thermal properties and weight loss (%) were determined, the degradation degree was not significant enough to affect the cast performance for 1 year. However, the results could be used to predict the multiyear degradation over time, suggesting that device removal surgery may not be necessary if the degradation property is further optimized. The SMP library did not present cytotoxicity when the CCK‐8 assay was conducted after the eluates of the 6‐arm 94% PCL – 06% PGMA were added to L929 cells in a series of dilutions (50%, 75%, and 100%) for one day (Figure S4, Supporting Information).

Vascular casts were produced using the 6‐arm 94% PCL – 06% PGMA by casting in a polydimethylsiloxane (PDMS) mold whose structure was generated using a 3D printed model, followed by two‐step crosslinking under UV (Figure S5a, Supporting Information). The original wrap shape was programmed to recover from a temporary plate shape, which facilitated deployment by covering the anastomosis part (Figure S5b, Supporting Information). As the 2nd crosslinking duration decreased, *T*_m_ was reduced to recover the shape around the body temperature due to a reduction in the crystallinity (Figure S5c, Supporting Information). The strand size of vascular cast was adjusted to 200 µm among the test sizes as the maximal strain was increased over 150%, thereby supporting the elastic synchronization with arterial contractility (Figure S5d, Supporting Information). The bridge addition increased the circumferential tensile strength to suppress vein dilation with the calculated fixity (Figure S5e, Supporting Information).

**Figure 3:**
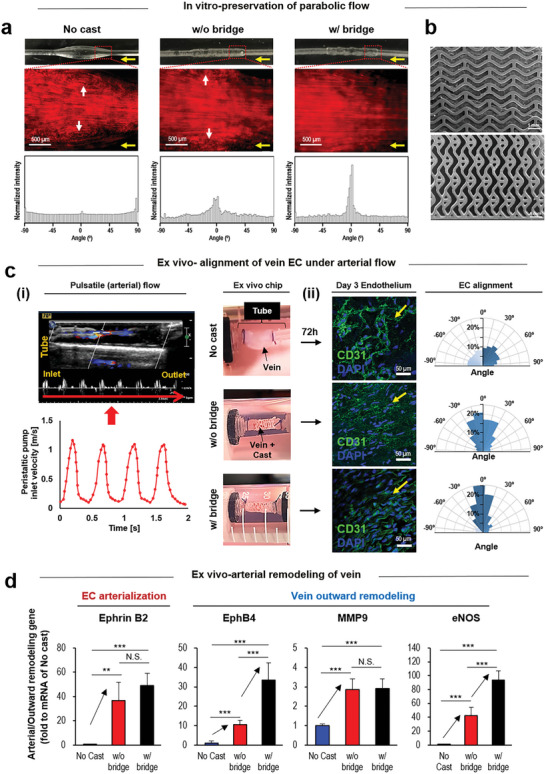
*Ex vivo* validation of bridge effects on the healthy vein responding to arterial flow. a) The bridge markedly supports the preservation of parabolic flow, as indicated by the particle angle distribution. The test groups were compared by visualizing particle flow through a 3D printed polydimethylsiloxane (PDMS) bioreactor that was designed following the CFD analysis of venous geometry in the systolic phase. (Yellow arrow: flow direction, / white arrow: disturbed flow). b) The beneficial effect was analyzed by comparing the w/o (top) versus w/ bridge (bottom) structure. c) As major characteristics of arterial hemodynamics, (i) the pulsatile flow velocity and pressure were generated into the ex‐vivo system of artery‐vein (AV) graft model in which the vein was connected with a silicone tube at each side to set the test groups (no cast, w/o bridge, and w/bridge). The inlet flow was provided by a peristaltic pump, as shown by doppler ultrasonic waves. (ii) The bridge helped the vein ECs to align with the flow direction most as determined through en face staining of each test group after 72 h of ex vivo culture, followed by the quantitative profiling of directionality with rose plotting. (yellow arrow: blood flow direction). d) The cast groups supported arterialization of the vein ECs, as evidenced by more expression of Ephrin (Eph) gene compared to no cast. Moreover, the bridge improved the cast effect on arterialization of the vein, as indicated by the higher level expression of EphB4, MMP, and eNOS genes compared to no cast on day 3 of the ex vivo perfusion. *p < 0.05, **p < 0.01, and ***p < 0.001 (*N* = 3).

The bridge addition effectively preserved the parabolic flow pattern as evidenced by the particle angle distribution (**Figure** **3**a). The test groups were compared by visualizing the particle flow using a 3D printed PDMS bioreactor that was designed based on CFD analysis of the venous geometry in the systolic phase. The beneficial effect was analyzed by comparing the w/o versus w/ bridge structure (Figure 3b). An ex vivo system was customized to examine vein dilation in response to the arterial hemodynamics (Figure S6, Supporting Information). The system was set up to generate arterial shear stress and pressure using a peristatic pump by controlling the flow of inner tube with adjusting the potential energy of media reservoir. As the height of media reservoir was elevated, the potential energy increased the venous pressure, which enabled the calculation of pressure. As major characteristics of the arterial hemodynamics (Figure 3c), the pulsatile flow velocity and pressure were generated into the ex vivo system of artery‐vein (AV) graft model in which a vein was connected to a silicone tube at each side in the test groups (no cast, w/o bridge, and w/bridge). The inlet flow was provided by a peristaltic pump as shown by the Doppler ultrasonic waves. The bridge addition helped vein ECs to align with the flow direction mostly as determined through en face staining of each test vein after 72 h of culture in the ex vivo system, followed by quantitative profiling of directionality using rose plotting. The cast groups supported arterialization of vein ECs, as demonstrated by the increased expression of Ephrin B2 (Ephrin B2) gene compared to no cast group (Figure 3d). Moreover, the bridge addition improved the cast effects on the healthy vein functions, as supported by the higher expression levels of EphB4, MMP, and eNOS genes compared to no cast group on day 3 following the post‐ex vivo perfusion.^[^ ^16^ ^]^

**Figure 4:**
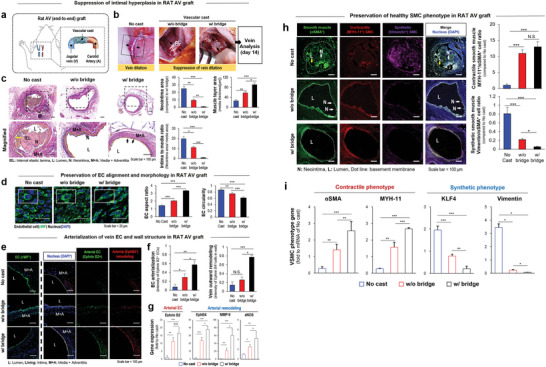
Rat model validation of the advantageous bridge effects on AV grafting. a) In rats, a carotid artery is grafted to the external jugular vein (AV graft), and the vascular cast is deployed to wrap the AV graft by covering the anastomosis site. b) Vein dilation (no cast) is visibly suppressed by w/o bridge and furthermore with w/ bridge; and the veins were subjected to follow‐up analyses at day 14 post grafting. c) The bridge supported the cast function to prevent the intimal hyperplasia of veins most effectively, as demonstrated in the comparisons of test groups by Verhoeff‐van Gieson staining of the vein cross‐sections along with a quantitative analysis of the neointima area, media thickness, and intima to media ratio. d) As a clear indication to preserve arterial flow, the bridge promoted the cast effects to significantly induce EC alignment upon comparison with the other groups by en face staining of the test veins. The aligned EC maintained the flow directional elongation with tight junction each other, thereby increasing the aspect ratio by reducing the circularity, as shown in the quantitative analyses of EC morphology. e) The advantageous bridge effects were shown in promoting the arterial marker expression (Eph B2) of vein ECs (vWF^+^) with the remodeling marker (EphB4) at the protein levels from confocal imaging with f) a quantitative analysis. g) The results were further supported by the marker gene expression of arterial EC (Ephrin B2) and arterialization of the vein (EphB4, MMP9, and eNOS). h) The bridge also promoted the cast effect to maintain the healthy contractile phenotype (MYH‐11) of vein SMCs (*α*SMA^+^) with the reduced expression of the pathological phenotype marker (Vimentin) at the protein levels by confocal imaging with a quantitative analysis. i) Compared to no cast group, the bridge further enhanced the pro‐contractile effect of vascular cast on the SMC phenotype, as evidenced by the most upregulated expression of contractile marker genes (*α*SMA and MYH‐11) as opposed to the synthetic marker genes (KLF4 and Vimentin). *p < 0.05, **p < 0.01, and ***p < 0.001 (*N* = 4).

A rat model was used to validate the advantageous bridge effects on AV grafting. A carotid artery was grafted to an external jugular vein (AV graft); and the vascular cast wrapped the AV graft by covering the anastomosis site (**Figure** **4**a).^[^ ^17^ ^]^ Vein dilation (in no cast group) was visibly suppressed, as evidenced in the w/o bridge group, and even more so in the w/ bridge group; and the veins were subjected to the follow‐up analyses at day 14 postgrafting (Figure 4b). The bridge addition most effectively supported the cast function to prevent intimal hyperplasia of veins, as evidenced by Verhoeff‐van Gieson staining of the vein cross‐sections with a quantitative analysis of the neointimal area, media thickness, and intima‐to‐media ratio in the test groups (Figure 4c).^[^ ^18^ ^]^ As a clear indication to preserve arterial flow (Figure 4d), the bridge addition promoted the cast effects in inducing EC alignment significantly when compared to the other groups using en face staining of test veins. The flow directional alignment of ECs supported the maintenance of the tight junctions with increased aspect ratio and the reduced circularity, as observed in the quantitative analyses of cell morphology.^[^ ^5^ ^]^ The advantageous bridge effects were further demonstrated in the quantitative analyses of confocal images (Figure 4e,f) where the arterial marker expression (EphB2) of vein ECs (vWF^+^) was promoted with an arterialization marker (EphB4) at the protein levels. These results were further supported by the marker gene expression of arterial EC (EphrinB2)^[^ ^6^ ^]^ and arterialization of the vein (EphB4, MMP9, eNOS) (Figure 4g). While CD31 was used as an EC marker in the ex vivo model (Figure 3c), vWF was used to identify ECs in this rabbit model to avoid overlapped interpretation by the CD31 expression of prevalent monocytes as reported previously.^[^ ^19^ ^]^ The combination of marker expression (vWF, EphrinB2&4, MMP9, and eNOS) with the aligned morphology aided the identification of EC behavior collaboratively. The bridge also promoted the cast effects to maintain the healthy contractile phenotype (MYH‐11) of venous SMCs (*α*SMA^+^) with a reduced expression of the pathological phenotype marker (Vimentin) at the protein levels as shown in the quantitative analysis of confocal imaging (Figure 4h).^[^ ^20^ ^]^ The MYH‐11^+^ signals in the lumen of the no cast group appeared to be artifact as this signal was absent in another set of images which further supported the results (Figure S7, Supporting Information). Compared to the no cast usage, the bridge further enhanced the pro‐contractile effect of vascular cast on the SMC phenotype as opposed to the synthetic marker genes (KLF4 and Vimentin)^[^ ^20^ ^]^ (Figure 4i), which was evidenced by most upregulated expression of contractile marker genes (*α*SMA and MYH‐11).

**Figure 5:**
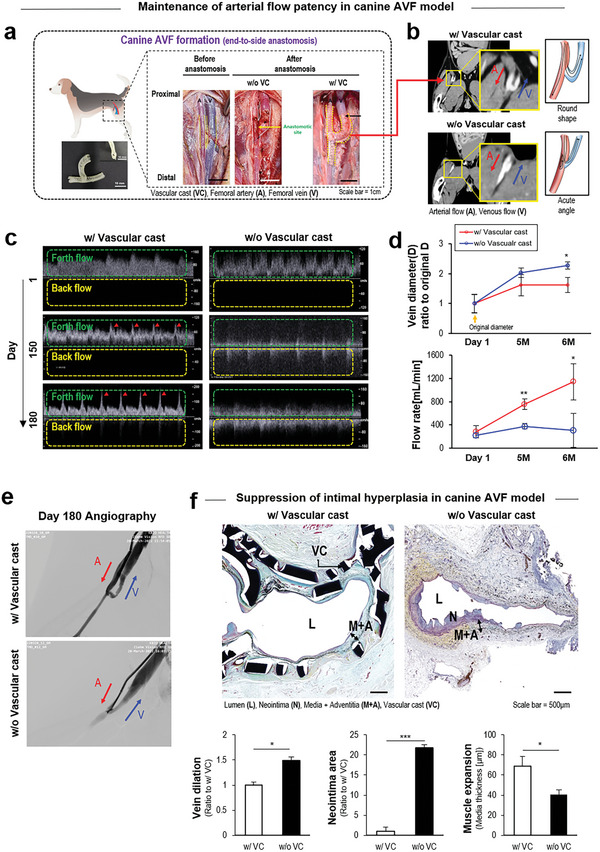
Canine AVF model with 6‐month validation of vascular cast advantages. a) Canine femoral vein is grafted to artery in an end‐to‐side fashion to model arteriovenous fistula (AVF). After U‐shape programming, vascular cast (w/ bridge) wraps the AVF by covering the anastomosis site for 6 months. b) The contrast imaging of Abdomen‐Pelvic computer tomography confirms facilitation of AVF patency by vascular cast with maintenance of the U‐shape blood flow. c) During the 6 months of cast deployment, doppler ultrasound analysis validates preservation of arterial flow patency by vascular cast (red arrow: arterial pulsatile flow) in contrast to the flow disturbance seen in w/o vascular cast (yellow box: back flow). d) The doppler results were quantitatively analyzed to compare the vein diameter over time and associated flow rate between w/ and w/o vascular cast. e) The 6‐month patency by vascular cast is further confirmed by angiography. f) Vascular cast suppresses intimal hyperplasia by supporting arterialization of the vein in pentachrome staining (elastic fibers and nuclei, black; collagen, yellow; mucin, light blue; muscle, red) with a quantitative image analysis. *p < 0.05, **p < 0.01, and ***p < 0.001 (*N* = 3 dogs).

Next, a canine model of arteriovenous fistula (AVF) was used for the 6‐month validation of vascular cast advantages. A canine femoral vein was grafted to an artery in an end‐to‐side fashion to produce an AVF model^[^ ^21^ ^]^ (**Figure** **5**a). After U‐shape programming, the vascular cast (w/ bridge) wrapped the AVF by covering the anastomosis site for 6 months. The contrast imaging of Abdomen‐Pelvic computer tomography confirmed the maintenance of AVF patency by the vascular cast with guiding the U‐shape blood flow (Figure 5b). During the 6 months of cast deployment, the Doppler ultrasound analysis validated the preservation of arterial flow patency by the vascular cast in contrast to the flow disturbance observed in w/o vascular cast (Figure 5c). The Doppler results were analyzed to calculate the vein diameter over time and associated flow rate of each test group (Figure 5d). The deployment of vascular cast suppressed vein dilation and thereby promoted the flow rate over 600 mL min^−1^ for 6 months, indicating that the improved flow patency was sufficient for hemodialysis access. The 6‐month patency by the vascular cast was further confirmed by angiography as there was no flow cessation shown, in contrast to w/o vascular cast (Figure 5e). The vascular cast suppressed intimal hyperplasia by supporting arterialization of the vein, as shown by pentachrome staining (Figure 5f).

## 3 Discussion

The pin‐point attack by a missile can result in the collapsing of entire structure whereas one finger support can save the collapsing of whole dam by relieving the stress concentration to distribute over the structure. Identification of the point to attack or support requires computational precalculation and experimental validation, which was successfully applied to this convergent study in adding bridges into the cast design. The successful outcome results in a series of advantages in a cascade fashion, as starting from improved elastic fixity to preservation of arterial flow profiles, and finally to venous arterialization in ECs, SMCs and wall remodeling. These advantages indicate many advancements of the present approach compared to the existing devices in the field, particularly in the following four aspects. First, while most existing devices address only a single effect of either suppression of vein dilation or expansion of stenotic vessels, the cast suppresses both vein dilation in arterial grafting and contractile synchronization arterial flow simultaneously. Second, the foundation of cascade events is that the SMP‐driven elastic fixity was enforced by adding the bridge components to the cast design, indicating the unique material benefit in utilizing the structure–function relationship. Third, the beneficial effects led to preservation of not only pulsatile laminar profiles but also the helical pattern, which maximize the efficiency of oxygen and nutrients delivery into the vascular wall while minimizing accumulation of thrombotic components and oxidized LDL.^[^ ^22^ ^]^ Finally, the vein arterialization was guided to promote media thickening towards adventitia with arterial phenotypic changes of ECs and SMCs.

To elaborate further the impactful effect of the cast deployment, the induction of helical (i.e., swirling and twisting) flow formation is evidenced by the contractile phenotype of SMCs with the increased thickness of media (Figures 4c,h‐I and 5f). These results supported the computational analyses of flow helicity (Figure 2c) because sufficient supplies of oxygen and nutrients to be delivered to the vascular wall was required to maintain the healthy actions of SMCs. Helical flow formation in the mid‐large size vessels promotes the transport efficiency of oxygen and nutrients to the vascular wall. The disturbed flow increases its interaction time with the vascular wall resulting in an accumulation of thrombotic components and oxidized LDL because of oscillatory movements with slow velocities.^[^ ^23^ ^]^ In contrast, the helical flow accelerates the repetition of short interactive circulation and thereby increases the chance to transport fresh contents, resulting in the prevention of pathological events particularly in vessels of curved or branched geometry.^[^ ^22^ ^]^ Hence, the helical flow formation is known to suppress the pathological flow formation.^[^ ^24^ ^]^ The increased perfusion on the vascular wall with mid‐large diameters promotes the efficiency of blood transport to neighboring tissue and small vasculatures. As an example, the helical flow formation in the aorta promoted perfusion on its neighboring and peripheral organs.^[^ ^25^ ^]^ Moreover, the absence of helical flow aggravated atherosclerosis and reduced renal perfusion in patients under vascular disease and renal artery stenosis, respectively.^[^ ^26^ ^]^ This effect represents an unprecedented benefit of the cast deployment, which can be considered as an significant advancement from existing devices in the field of cardiovascular medicine.

The venous arterialization at the cell level provides a new insight in the field of vascular biology because the preservation of arterial flow descriptors induced venous ECs to express arterial EC markers with promoting the contractile marker expression in SMCs. These collaborative effects led to the arterialization of veins due to the reduction of the property mismatch between veins and arteries in their end‐to‐end and side‐to‐end grafting in the rat and canine models, respectively. Based on the action–reaction phenomenon, the arterial hemodynamic environment activated the cell plasticity to respond to the changed environment more properly;^[^ ^27^ ^]^ thus, the adapted cell phenotypes supported effectively the preservation of arterial flow descriptors in turn. These results provide considerable insight into the vascular pathophysiological processes associated with the hemodynamic profiles, including flow disturbances and type change. Therefore, it is important to recognize that collaborations between engineers, scientists, and clinicians are required to establish a new therapeutic paradigm.

Computational modeling of both solid and flow simultaneously allowed for the prescreening of structure–function relationship.^[^ ^28^ ^]^ The input parameters were determined based on the mechanical property analyses of material and vessels, while the boundary conditions were set by considering blood pressure, AVF inlet velocity, peripheral resistance, and material properties.^[^ ^29^ ^]^ The cast effect was analyzed in considering of the vessel dilation caused by blood pressure and shear stress, allowing for a hemodynamic analysis to be performed using CFD modeling. This whole set of processes saved a substantial number of animal lives by reducing the scope of variables to be determined, suggesting an important guideline to avoid an empirical determination by relaying on subjective clinical opinions. Helical flow facilitates nutritional delivery to the vascular wall through its hemodynamic effects, which is demonstrated in clinical attempts to utilize the benefits.^[^ ^15a^ ^]^ This study suggests that the healthy blood flow (laminar and helical) should be preserved by the outside‐in control with reducing the mechanical mismatch between the artery and vein. This technical point was validated using a customized ex vivo system with rat and canine models. The ex vivo system provides a user‐specified control of each parameter, while the animal models serve as the two most popular models of vein‐to‐artery grafting, presenting different patterns of hemodynamic changes (end‐to‐end and side‐to‐end).^[^ ^17^, ^21^ ^]^ Together, this study proposes an efficient means to accelerate the preclinical process and thereby to improve the possibility of clinical success through the technological translation.

Despite the series of impactful points, further investigation is required when applying the bridge design to other devices including endovascular stents. Because the endovascular deployment can be facilitated by the shape programming for self‐expansion of the stent, and the bridge may induce healthy flow profiles even in the endovascular side. However, although the strut penetration into the vascular wall is fine‐controlled in current stent approaches, the inner diameter reduction by stent deployment often causes flow disturbances and consequently stenotic remodeling. Therefore, the external control from the outer wall side has been considered in a form of a vascular cast considering the important roles of outer wall factors including pericyte, vasa vasorum, and muscle expansion towards the adventitia side instead of the intimal side. The roles of pericytes and perivascular stem cells associated with vascular cast effects need to be investigated. Moreover, the causative role of EC arterialization in the contractile phenotype of SMCs has becomes an important topic to elucidate a new underlying mechanism of vascular biology.

While the stable wrapping position of vascular cast was observed in the adventitia area of rabbit end‐to‐end graft model (Figure 4c), the cast appeared at a position close to the intima area (i.e., right underneath the lumen) in the canine side‐to‐end graft model (Figure 5f). As the vein diameter and flow patency were maintained over time for 6 months (Figure 5d), the compression pressure caused by the cast might not be an issue. Also, the degradation should have a negligible effect on the performance of the cast because the SMP properties were not altered significantly for one year (Figure S3d, Supporting Information). Meanwhile, it was most likely that the staining process disturbed the cast position, but this result should motivate further consideration for upgrading the device design and deployment method to maintain some space between the cast and vessel wall using an antiadhesive gel. The vascular cast represents a convergent technology with a series of collaborations among the SMP, bridge design, computer simulation, hemodynamic control, cell phenotype, and tissue response. Each technical component provides an important insight to the relevant fields.

## Conflict of Interest

The authors declare no conflict of interest.

## Supporting information

Supporting information

## Data Availability

The data that support the findings of this study are available from the corresponding author upon reasonable request.
