# Supplementary material for: Vascular Cast to Program Antistenotic Hemodynamics and Remodeling of Vein Graft
Source: Adv Sci (Weinh). 2023 Feb 2;10(10):2204993. doi: 10.1002/advs.202204993 (PMC10074125; doi:10.1002/advs.202204993)
Supplement: Supplementary file 1 — Supporting information [file ADVS-10-2204993-s001.pdf]

ADVANCED SCIENCE Open Access

Supporting Information

for *Adv. Sci.*, DOI 10.1002/advs.202204993

Vascular Cast to Program Antistenotic Hemodynamics and Remodeling of Vein Graft

Hyunsu Ha, Ju Young Park, Chan Hee Lee, Deok-Hyeon Son, Soon Won Chung, Sewoom Baek, Kyubae Lee, Kang Suk Lee, Se Won Yi, Mi-Lan Kang, Dae-Hyun Kim and Hak-Joon Sung**

Supporting Information

Vascular cast to program anti-stenotic hemodynamics and remodeling of vein graft

Hyunsu Ha[§], Ju Young Park[§], Chan Hee Lee[§], Deok-Hyeon Son, Soon Won Chung, Sewoom Baek, Kyubae Lee, Kang Suk Lee, Se Won Yi, Mi-Lan Kang, Dae-Hyun Kim[*], and Hak-Joon Sung[*]

Experimental Section

Synthesis of shape memory polymers (SMPs)

A library of SMPs was synthesized via ring-opening polymerization by varying the molar ratio of ε-caprolactone (CL, Aladdin) to glycidyl methacrylate (GMA, Sigma-Aldrich). First, the initiator (dipentaerythritol, 0.5 mmol, Sigma-Aldrich) and inhibitor (HQ, 1/10 mmol of GMA, Sigma-Aldrich) were placed in a three-necked flask, followed by vacuum drying for 20 min. The distilled CL was injected into the flask under nitrogen purging, and the flask was submerged in an oil bath at 110 °C, followed by reaction with GMA after 10 min. A solution of 3.5 mmol 1,5,7-triazabicyclo [4.4.0] dec-5-ene (TBD, TCI) and acetonitrile (ACN, Sigma-Aldrich) was added to the flask and reacted for 6 h under nitrogen purging. The synthesized SMPs were dissolved in chloroform (Sigma-Aldrich), precipitated with cold ethyl ether (Samchun Chemicals), and dried for one day under a vacuum, thereby yielding a 6-arm x% PCL-*co*-y% PGMA (%: molar ratio).

SMP Characterization

The structure and molar ratio of the 6-arm x% PCL-*co*-y% PGMA were analyzed using ^{1}H-nuclear magnetic resonance (^{1}H-NMR, Avance III 400-MHz NMR spectrometer; Bruker Biospin, Billerica, USA) (Figure S3b). The molar ratio of PCL (x%) and PGMA (y%) was calculated as the area ratio of peak (δ =2.41 ppm, PCL) and peak (δ =6.13 ppm, PGMA). The thermal properties of the 6-arm

x% PCL-*co*-*y*% PGMA were determined using differential scanning calorimetry (DSC, DSC214; NETZSCH, Germany). The samples were heated from -50 ℃ to 150 ℃ at a rate of 10 ℃ min^{-1} in a nitrogen atmosphere. The melting temperature (T_m), crystallization temperature (T_c) and melting enthalpy (ΔH_m) were determined using DSC. The crystallinity (X_c) was calculated using the following equation (1):

$$X_c = \frac{\Delta H_c}{\Delta H_c^{\circ}} \times 100\% \qquad (1)$$

where the melting enthalpy of 100% crystalline PCL is 139.5 J g^{-1}.[1]

A 6-arm 94% PCL-*co*-06% PGMA was selected to produce the vascular cast owing to the shape recovery near 40 ℃. The mechanical properties of the 6-arm 94% PCL-*co*-06% PGMA were analyzed using a DMA (dynamic mechanical analyzer, Discovery DMA 850, TA instrument Inc. New Castle, DE, USA) in film form.

SMP degradation

The accelerated aging test was performed to determine SMP degradation following the American Society of Testing and Materials (ASTM) international standard 1980 and equations (2-4) as defined below to calculate the duration of real-world life-time (RT) equivalent usage.

$$\text{AAT} = \text{Desired (RT)} / \text{AAF} \qquad (2)$$

$$\text{AAF} = Q_{10}^{[(T_{AA}-T_{RT})/10]} \qquad (3)$$

$$Q_{10} = 2.0 \qquad (4)$$

AAT: Accelerated aging time

Desired (RT): Desired duration of RT equivalent usage

AAF: Accelerated aging factor (rate) = ratio of Desired (RT) to AAT

Q_{10}: Temperature coefficient = how quickly a material changes when the temperature is increased by +10°C

T_{AA}: Accelerated aging temperature

T_{RT}: 37°C

Through the calculation, the following values were obtained in correspondence to 12 months (5,6).

$$AAF = 2.0^{3.3} = 9.85 \quad (5)$$

$$AAT = 365/9.85 = 37.01 \quad (6)$$

Following the obtained values, the accelerated aging condition was set by incubating SMP samples in normal saline (pH 7.4) at 70 ± 2 ℃ for 37days. Then, degradation-mediated changes in the thermal properties were determined by DSC analysis. Accordingly, the weight loss (%) was calculated using the following equation (7):

$$\frac{m_0 - m_a}{m_0} \times 100\% \quad (7)$$

m_0 is initial mass, m_a is mass after degradation.

Elastic property characterization of rabbit vessels in a customized ex vivo system

The mechanical properties of the vessels were analyzed and entered for computer modeling as the input parameters. A rabbit infrarenal aorta and interior vena cava (IVC) were harvested and cut into 1 cm-long segments. All rabbit experiments and management procedures were approved by the Institutional Animal Care and Use Committee of the Yonsei Laboratory Animal Research Center (2021-0048). New Zealand white rabbits (male 3.0-3.5 kg, DooYeol Biotech, Seoul, Republic of Korea) were anesthetized by intramuscular injection of tiletamine-zolazepam (Zoletil 50; 10 mg kg^{-1}, Virbac Lab, Carros, France) until euthanizing using potassium chloride (KCl; 20 mg kg^{-1}, Choongwae Pharma Corporation, Seoul, Republic of Korea). After incision along the mid-line of the abdomen, the tissues around the aorta and IVC were dissected, and the artery and vein were harvested without vascular damage, followed by immediate storage in PBS (Welgene, Gyeongsangbuk-do, Republic of Korea).

The hyper-elastic properties of the ex vivo blood vessels were analyzed in a customized chamber (8022812, Festo, Seoul, Republic of Korea) by increasing the flow pressure. The rabbit artery and vein were connected to silicone tubes (SL-0102, LK LAB Korea, Namyangju, Republic of Korea) on each side using 6-0 silk sutures (W2814, Ethicon, Somerville, MA, USA) and straight connectors (30622-49, Donginbio, Seoul, Republic of Korea) in the pressure test chamber. The expansion of the diameter of each test vessel in response to incremental pressure changes was recorded. Subsequently, the relationship between the pressure and wall tension was analyzed by calculating the experimental data using Laplace's law ($\sigma = \Delta pt/ri$, where σ is the wall tension, Δp is the inner pressure-outer pressure, t is the wall thickness, and r is the radius of the cylinder).[2] The hyperelastic property values of the arteries and veins were entered as input data for computational modeling, and curve fitting was conducted using the Mooney–Rivlin 2 parameter model.[3]

Structural modeling

The 2D and 3D models of vascular cast were produced using Fusion 360 CAD programs (version 2018, Autodesk, California, USA). The base design of the strand structure was derived from polymeric stents and altered without (w/o) and with (w/) bridge, as the bridge structure was added to relieve the stress from vein dilation. Structural modeling was conducted using ANSYS Mechanical software (ANSYS 2020R1, ANSYS, Canonsburg, PA, USA) by inputting the mechanical property values of SMP, arteries, and veins that were experimentally obtained, as previously indicated. Structural modeling enabled the simulation of the bridge effect when the cast was deployed to wrap the artery-vein-artery graft by analyzing the structures of vessel and device in response to arterial pressure and pulsative blood flow. The diameter and length of each artery and vein were set to 3 and 30 mm, respectively, while the device length was set to 25 mm to cover the entire anastomosis and the proximal and distal parts of graft. The thickness and length of cast strands were set to 400 μm and 200 μm, respectively. The von-mises stress against the deformation

of vascular cast and vein was monitored, and the elastic changes of device were analyzed with increased flow pressure.

Computational fluid dynamics (CFD) modeling

The structure of blood vessels changes in response to blood flow and pressure, which alters the flow profiles. The flow parameters were analyzed by CFD modeling using ANSYS Fluent software (ANSYS 2020R1, ANSYS, Canonsburg, PA, USA). The vessel geometry was constructed by entering the structural modeling data and then discretizing them into mesh structures through a finite element method (FEM). The blood was assumed to be a highly viscous non-Newtonian fluid at a low shear rate, and the viscosity decreases upon increasing the shear rate. Next, the Carreau-Yasuda model was applied (viscosity at zero shear rate = 0.056 Pa·s, viscosity at infinite shear rate = 0.00345 Pa·s, time constant = 1.902 s, power law index = 0.22, Yasuda exponent = 1.25).[4] The blood density was 1.060 kg m^{-3}, which was assumed to be incompressible. Three groups (no cast, w/o bridge, and w/bridge) were used in the model.

As a boundary condition, the vessel wall was considered to be no-slip, and the blood flow and pressure at the arterial inlet were obtained from the clinical AVF data.[5] Modeling was conducted under transient conditions with a time interval of 0.001 s, and 400 iterations for each time step. The hemodynamic descriptor values were obtained after processing the modeling results by programming the user-defined functions. The descriptors included the time-averaged wall shear stress (TAWSS), oscillatory shear index (OSI), average helical intensity (h2), and localized normal health (LNH). The anti-flow disturbance effect of vascular cast was revealed through a streamline analysis with quantification of the diastolic wall shear stress (WSSd), TAWSS, and OSI. In addition, the helical flow formation was analyzed as a healthy flow characteristic by quantifying the average helicity intensity and secondary velocity, whose vector is perpendicular to the vessel axis as an inducer of helical flow.[6]

Vascular cast fabrication

The computer-aided design (CAD) of vascular cast was first generated using the Solidworks software (Solidworks 2021, Dassault system, Vélizzy-Villacoublay, France). The flat shape (2D type) of cast model was then 3D-printed (IM2, Carima, Seoul, Republic of Korea) to generate a modeling structure in PDMS. SMP (6arm 94%PCL-*co*-06%PGMA) was dissolved in *N*-methyl-2-pyrrolidone (NMP, Sigma-Aldrich, 1 g mL^{-1}), and 1% photo-initiator (Irgacure 2959, Sigma-Aldrich) was added to the solution. The SMP solution was placed in the PDMS mold, pressed with glass, and then subjected to the first crosslinking using an ultraviolet lamp (300–400 nm, UVACUBE 400; Hoenle, Germany) for 30 s. After separating the structure from the glass, a cylindrical shape was formed, followed by a second crosslinking step for 20 min. The cast was washed with distilled water, dried under a vacuum, and stored at room temperature until further use.

Thermomechanical properties

The thermal properties of vascular cast were characterized by DSC (DSC214; NETZSCH, Germany). The tensile strength and Young's modulus were determined by varying the strand size (200, 300, and 400 μm) or adding the bridge structure in a flat 2D printed structure using DMA (DMA 850, TA instrument Inc. New Castle, DE, USA) under 10 % min-1 of the strain rate at 37 °C. The surface properties were characterized by field-emission scanning electron microscopy (FE-SEM; MERLIN, Zeiss, Oberkochen, Baden-Wüttemberg, Germany) after coating with platinum.

Cytotoxicity

SMP (6-arm 94% PCL-06% PGMA) films were prepared having a width of 4 cm, height of 1 cm, and thickness of 0.4 cm, and were eluted in MEM medium (SH30024, HyClone, Logan, UT, USA) with 1 g per 5 mL for 72 h. The cytotoxicity of test films on mouse fibroblasts (L929) was determined using a cell counting kit-8 (CCK-8) assay (CK04, Dojindo, MD, USA) according to

ISO 10993-5. L929 cells were seeded in a 96 well-plate (30096, SPL, Kyonggi-do, Republic of Korea) at a density of 5×10^4 cells per well for 24 h. The elution medium was diluted to set the test concentrations (50, 75, and 100 %) and incubated with L929 cells for 72 h. The cells were then treated with CCK-8 solution for 2 h at 37 °C, followed by absorbance reading at 450 nm in a microplate reader (A51119600C, Thermo Scientific, MA, USA) and normalizing each readout to that of the non-treated group.

Particle flow visualization

The geometry of the blood vessel in the systolic phase was constructed using CFD modeling and 3D printing (Raised3D Pro2, Irvine, CA, USA), which was used to generate the vessel structure in a poly(dimethylsiloxane) (PDMS) mold as a flow chamber after dissolving the 3D printed structure using tetrahydroflurane (THF). The flow chamber was coated with Pluronic F-127 (1 w/v%, P2443, Sigma-Aldrich) to prevent particle adhesion. The flow was visualized by perfusing red polystyrene microspheres (diameter: 4 µm, Invitrogen, Carlsbad, CA) using a peristaltic pump (BT100-1L, LongerPump, Amerham, UK) under a microscope. The particle images were analyzed using the fast Fourier transform (FFT) function of the Image J software (National Institution of Health, Bethesda, MD, USA), followed by determining the angle distribution of the blood flow direction using a plug-in option.

Ex vivo system

A 3D *ex vivo* culture system was constructed to vary the typical arterial pressure (60–100 mmHg) and shear rate (10-15 dyne cm^{-2}) from the artery-vein grafting.[5] A chamber (3.3 length × 1.5 width × 2 cm) was produced by 3D printing (Raised3D Pro2, Irvine, CA, USA) polylactic acid (PLA; Raise 3D, Irvine, CA, USA). In the chamber, a segment (2 cm) of each rabbit jugular vein with a cast (w/o or w/ bridge) or no cast was tied with male/female Luers (O.D. = 1.6 mm; 45518-00/45508-00, Cole-Parmer, Vernon Hills, IL, USA) using 4-0 Vicryl sutures. The chamber was then

filled with endothelial cell growth medium-2 (EGM-2; CC-3162/CC4176, Lonza, Basel, Switzerland) and connected to a peristaltic pump (BT100-1L, LongerPump, Amersham, UK) using silicone tubes (I.D. = 1 mm, O.D. = 2 mm; SL-0102, LK LAB Korea, Namyangju, Republic of Korea). Under a closed circulation, the pressure was controlled by varying the potential energy (height difference, Δh) and viscosity, as calculated using *Bernoulli's equation* ($\Delta P= \rho \cdot g \cdot \Delta h$, where P, ρ, and g indicate the pressure, fluid density, and acceleration due to gravity, respectively) and *Hagen–Poiseuille's equation* ($\Delta p= 8\mu LQ\pi R3$). Arterial shear stress (10-15 dyne cm^{-2}) was generated by controlling the pulsatile flow rate, as calculated using *Poiseuille's equation* ($\tau= 4\mu Q\pi R3$, where τ, R, μ, and Q indicate the shear stress, vein radius, fluid viscosity, and flow rate, respectively). Doppler sonographic imaging (iU22 xMatrix DS, Philips, Amsterdam, Netherlands) was used to confirm the arterial shear stress and pulse rate by applying a flow rate of 8000 μL min^{-1}, a pulse rate of 125 min^{-1}, a maximum viscosity of 1.18 m s^{-1}, and shear stress of 12.63 dyne cm^{-2}, followed by culturing each vein for 3 days at 37 °C.

En face staining

Following the ex vivo culturing process, each vein was rinsed with phosphate buffer saline (PBS; 1X, pH 7.4, Welgene, Gyeongsangbuk-do, Republic of Korea) and then fixed with 4% paraformaldehyde (CellNest, Gyeonggi-do, Republic of Korea) for 24 h. The vein samples were then permeabilized with 0.1% Triton X-100 (Sigma-Aldrich) in PBS and blocked with 5% bovine serum albumin (BSA; 82-100-6, Millipore, Burlington, MA, USA) at room temperature for 2 h. The samples were then incubated with primary antibodies against CD31 (1:100, NB600-562, Novus, St. Charles, MO, USA) and for the anti-Von Willebrand Factor (1:100, ab6994, Abcam, Cambridge, Cambridge, UK) overnight at 4 °C. The samples were washed and incubated with secondary antibodies (1:1000, Alexa Fluor 488; 115-095-003 and 111-545-003, Jackson ImmunoResearch Laboratories, West Grove, PA, USA) for 2 h in the dark. Nuclei were counterstained with NucBlue Live ReadyProbes Reagent (Invitrogen, Waltham, MA, USA), followed by confocal imaging (LSM

780; Zeiss, Oberkochen, Land Baden-Württemberg, Germany). The morphology and aspect ratio of ECs were analyzed using a rose plot.

Rat artery-vein (AV) graft model

All animal experiments were approved by the Institutional Animal Care and Use Committee (IACUC) of the Yonsei Laboratory Animal Research Center (YLARC) (permit number 2022-0080). An AV graft was modeled in SD rats (female, 12 weeks old, Orient Bio, Seoul, Republic of Korea) through end-to-end anastomosis between the common carotid artery and the external jugular vein.[7] The rats were anesthetized by intramuscular injection of Zoletil (50 mg kg^{-1}, Zoletil™, Virbac Korea, Seoul, Republic of Korea) and xylazine (10 mg kg^{-1}, Rompun®, Bayer Korea, Seoul, Republic of Korea) into the thigh muscle. During the operation, respiratory anesthesia with isoflurane (< 2.5 %, Piramal, PA, USA) was maintained.

The cervical area was shaved and sterilized using povidone-iodine (Green Pharmaceutical, Jincheon, Republic of Korea), followed by a 2 cm incision along the median line of the trachea. After dissecting the adipose tissue surrounding the right external jugular vein, tiny branches of the jugular vein were ligated using 10-0 Ethilon (W2814, Ethicon). The distal vein was ligated using a 5-0 Vicryl suture (W9761), and the proximal vein was clamped using a microvascular vein clamp (S&T Vascular clamp, 00396, Switzerland). A midline incision on the sternohyoid muscle was then made, the carotid artery next to the trachea was exposed, and the surrounding tissues were dissected. Subsequently, the vein between the clamps was excised under irritation with normal saline (Choongwae Pharma Corporation, Seoul, Republic of Korea) and heparin (10 IU mL^{-1}, Choongwae Pharma Corporation, Seoul, Republic of Korea). The carotid artery was then exposed through a midline incision on the rat sternohyoid muscle, followed by dissection of the surrounding tissues. The distal artery was sutured with a 5-0 Vicryl suture, the proximal artery was clamped using a microvascular artery clamp (JD-S-101MC-03, JEUNGDO, Republic of Korea), and the

artery between the clamps was excised with saline irrigation. A triangular space was created by widening the space between the hyoid, sternohyoid, and sternomastoid muscles using mosquito forceps. The carotid artery was pulled out to this space for device deployment (cast with or without bridge: 1 mm inner diameter and 5 mm length).

The device groups were sterilized using ethylene oxide (EO) gas and stored at room temperature. The carotid artery was passed through the vascular cast, the device was moved to the jugular vein, and end-to-end anastomosis was performed using a 10-0 Ethilon suture (Ethicon). The vein was then released from the clamp, the arterial clamp was released to check the patency, and the ability to control the vein dilation was observed. The muscle and subcutaneous tissue layers were sutured using 5-0 Vicryl (Ethicon), and the skin layer was sutured using 5-0 Ethilon (W1661G, Ethicon). The rats were monitored daily for two weeks until they were euthanized to obtain AV graft specimens.

Canine AV fistula (AVF) model

All animal experiments were approved by the IACUC of the Osong Medical Innovation Foundation (KBIO health), (Permit No. KBIO-IACUC-2021-174). Female beagle dogs (7- 8 kg, Orientbio, Seong-Nam, Republic of Korea) were anesthetized by intramuscular injection of tiletamine-zolazepam (5 mg kg^{-1}, Zoletil™, Virbac Korea) and xylazine (2 mg kg^{-1}, Rompun®, Bayer Korea), which were maintained by endotracheal intubation with isoflurane (< 2.5 %, Piramal) during surgery.

The AVF was modeled in the right hindlimb (N = 6, each group) by a midline incision (approximately 5 cm) at the right inner thigh, followed by exposure of the femoral artery and vein via a blunt dissection of the underlying connective tissues under intravenous heparin injection (200 IU kg^{-1}). The distal end of femoral vein was ligated using 4-0 silk (BLACK SILK, Ailee Co., Ltd.) and cut. The proximal and distal parts of femoral artery were temporally occluded using a vascular

tourniquet. Finally, a longitudinal incision was made into the side wall of femoral artery (approximately 5 mm), which was followed by end-to-side anastomosis through suturing with 7-0 prolene (Surgipro™ II, Covidien™, Medtronic, Minneapolis, USA). The cut end of the vein was placed into the cast and anastomosed with the artery to wrap the entire AV graft, followed by closing the subcutaneous layer and skin. The diameters of femoral vein and artery (proximal and distal) were measured weekly using ultrasonography (EKO 7, Samsung Medison Co., Ltd., Republic of Korea). The pulse wave (PW) doppler mode of ultrasonography was quantitatively analyzed to obtain the velocity profile of blood flow. The vessel cross-sectional area was used to calculate the vein diameter, which was then used with the mean velocity to analyze the blood flow rate. After 6 months, a catheter-based angiography was performed to confirm the AVF shape and patency. Briefly, the beagles were anesthetized as described above and placed in a dorsal recumbent position. The left carotid artery was exposed via a cervical ventral midline incision, and a vascular sheath (6 Fr, 6F ENVOY DA catheter; Codman, MA, USA) was inserted. An angiographic catheter (6 Fr) was then placed at the right iliac artery via the vascular sheath. An angiography was performed with a 5 mL kg^{-1} of iohexol (Omnipaque ™, 300 mg I mL-^{1}) bolus injection using a C-arm (Ziehm Vision RFD, 3D, Ziehm imaging, Nuremberg, Germany). Computed tomography angiography (CTA) (16-channel multi-detector; BrightSpeed Elite, GE Healthcare, Fairfield, CT, USA) was also performed with an intravenous injection of 2 ml/kg iohexol to analyze the 3D vascular structures. The beagles were euthanized by a bolus intravenous injection of potassium chloride (KCl; 20 mg kg^{-1}; Choongwae Pharma Corporation, Seoul, Republic of Korea) under general anesthesia, followed by harvesting the AVF tissue at the surgical site of each group for further analyses.

Histological and immunohistochemical analyses

In vivo tissue samples were rinsed with PBS and fixed with 4% paraformaldehyde (CellNest, Gyeonggi-do, Republic of Korea) for 24 h. The samples were embedded in paraffin wax, cross-sectioned into 5 μm slices and stained with hematoxylin-eosin and Verhoeff-van Gieson staining.

Tissue slides were subjected to immunofluorescence staining by deparaffinization three times with xylene (Duksan, Gyeonggi-do, Republic of Korea) and rehydration in ethanol (Duksan) by serial incubation (100 %, 95 %, 80 %, 70 % (v/v) in distilled water). The slides were treated with citrate buffer (1X, pH 6.0, Sigma-Aldrich) to retrieve the antigens by heating for 30 min at 95 ℃, followed by blocking with 0.1 % Triton X-100 (Sigma-Aldrich) and 5 % bovine serum albumin (BSA; 82-100-6, Millipore) in PBS at room temperature for 2 h. The slides were then incubated with primary antibodies of the anti-Von Willebrand factor (1:100, ab6994, Abcam), ephrin B2 (1:100, Ls-B16452, Lsbio, Seattke, WA, USA), ephrin B4 (1:50, 2088-1-AP, Proteintech , Rosamond, IL, USA), α-SMA (1:1000, NB300-978, Novus, St. Charles, MO, USA), MYH-11 (1:200, NBP2-44532, Novus), and vimentin (1:200, NBP-44832, Novus) overnight at 4 ℃. The samples were washed and incubated with secondary antibodies: anti-rabbit Alexa Fluor 488 and 594 (1:1000, 111-545-003 and 111-585-003, Jackson ImmunoResearch Laboratories), anti-goat Alexa Fluor 488 (1:1000, A11055, Invitrogen, Waltham, MA, USA), and anti-mouse Alexa Fluor 594 (1:1000, 115-585-003, Jackson ImmunoResearch Laboratories) for 2 h in the dark. The nuclei were counterstained with NucBlue Live ReadyProbes Reagent (Invitrogen), followed by imaging using confocal laser scanning microscope (LSM 780).

Gene expression

In vivo tissues were processed to determine the marker gene expression of arterialization in the ECs (ephrin B2), vein (EphB4, MMP-9, and eNOS), and SMC phenotype (αSMA, MYH-11, KLF4, and vimentin). The total RNA was extracted using an RNA extraction kit (74106, Qiagen, Hilden, Germany) following the manufacturer protocol, and the RNA concentration was determined using a

Nanodrop 2000 spectrophotometer (ND2000, Thermo Fischer). Complementary(C) DNA was synthesized by reverse transcription using the AccuPower CycleScript RT premix (K2044, Bioneer). Real-time PCR (StepOne V2.3, Applied Biosystems, MA, USA) was run with cDNA using SYBR green and primers (**Table S2**, Supporting Information) through 40 cycles of the target gene amplification, with a holding stage at 95 °C for 10 min, denaturation stage at 95 °C for 1 min, and annealing stage at 60 °C for 1 min. Glyceraldehyde 3-phosphage dehydrogenase (GAPDH) was used as a housekeeping gene, and the relative gene expression was analyzed using the $2^{-\Delta CCt}$ method.

Statistical Analysis

All statistical analyses were conducted using Excel and Sigmaplot (V12.0, Systat Software, CA, USA); the data are presented as the mean ± standard deviation (SD) using at least three samples. The statistical significance was determined using an unpaired Student's t-test for two-group comparisons and a one-way analysis of variance (ANOVA) with Bonferroni's and Turkey's post hoc analysis for more than two group comparisons. *P*-values ($*p < 0.05$, $**p < 0.01$, and $***p < 0.001$) were considered statistically significant.

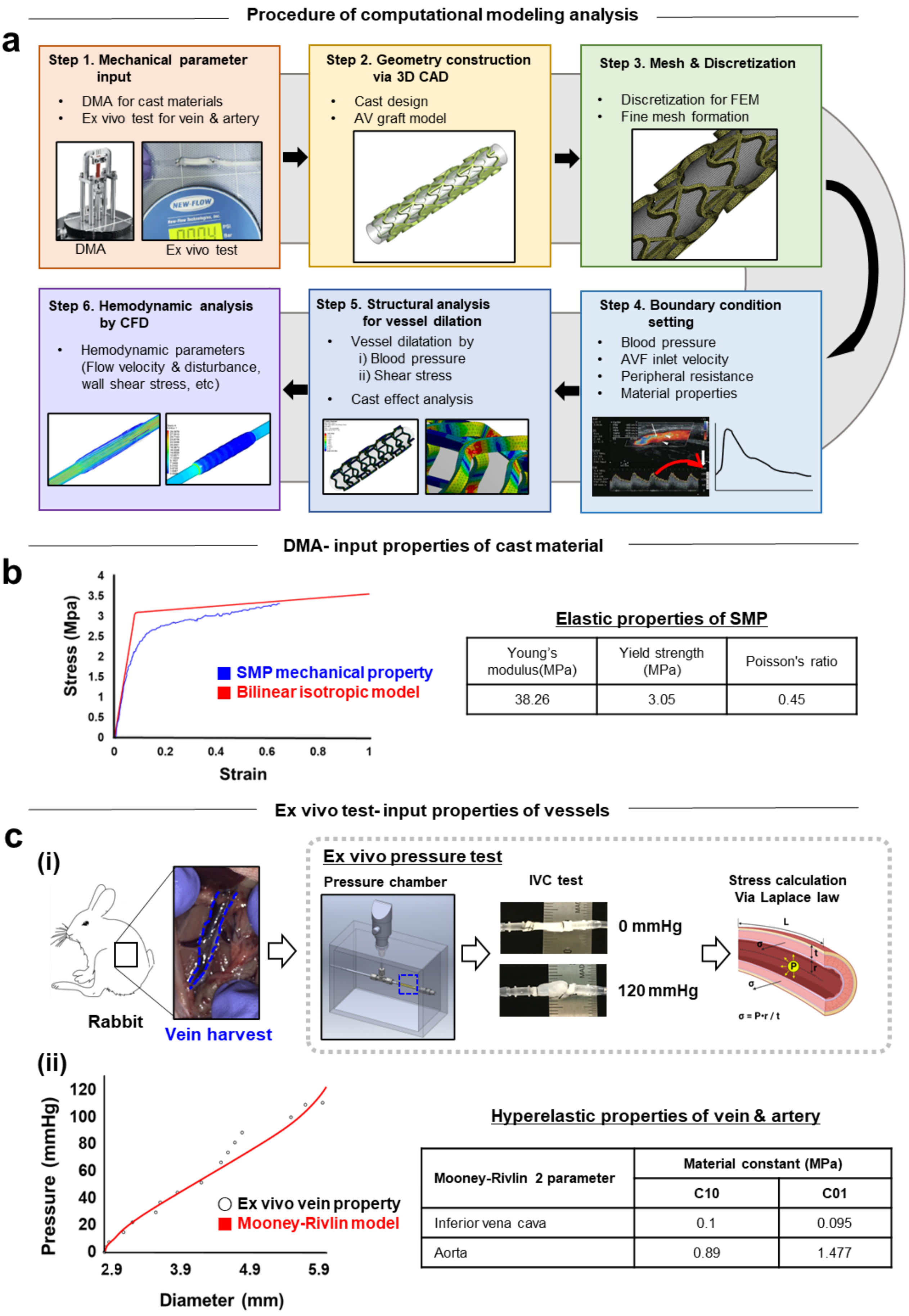

Young's modulus(MPa)	Yield strength (MPa)	Poisson's ratio
38.26	3.05	0.45

Mooney-Rivlin 2 parameter	Material constant (MPa)	
	C10	C01
Inferior vena cava	0.1	0.095
Aorta	0.89	1.477

Figure S1. Co-computational modeling of the vessel structure and hemodynamics with input

properties. **a)** The step-by-step procedure is set-up for co-computational modeling of solid structural dilation and fluid dynamics including the mechanical parameter, geometry construction by 3D CAD, meshing and discretization, and structural and CFD analyses. **b)** DMA is used to analyze the elastic property of SMP film using a bilinear isotropic model as a reference, and the elastic parameter values are input for the computational modeling analysis. **c)** The inferior vena cava (IVC) and aorta are harvested from rabbits, and the ex vivo system is set to calculate the vascular wall stress to the incremental pressure using Laplace law as a model for vein dilation. Consequently, the hyper-elastic parameter values of the vascular property are analyzed using the Mooney-Rivlin model as a reference, and input for the computer modelling analysis.

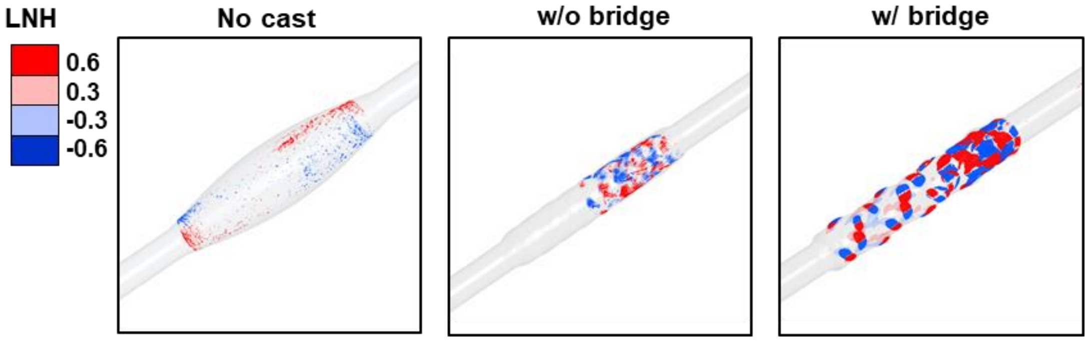

Figure S2. Improvement of local normalized helicity (LNH) by adding a bridge to the vascular cast. The addition of the bridge further promoted the cast effect on the induction of healthy helical flow, as indicted by the marked increases of LNH compared to that with no cast.

a **SMP synthesis process**

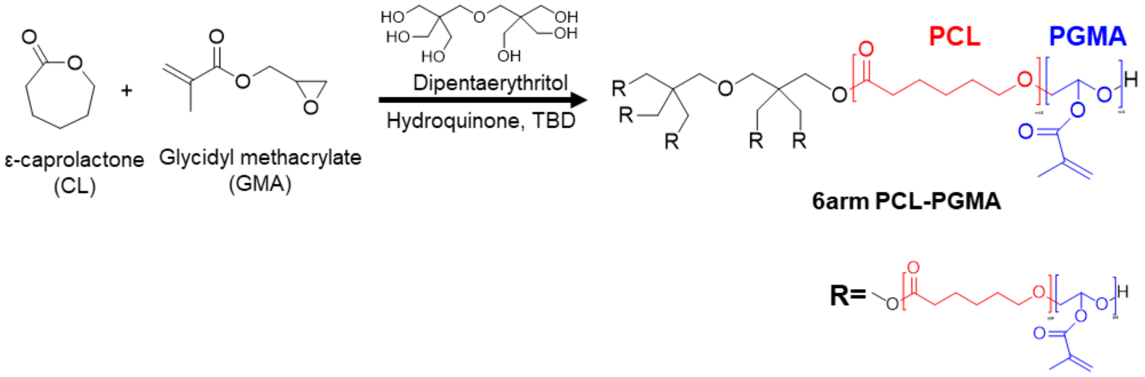

b **SMP synthesis validation**

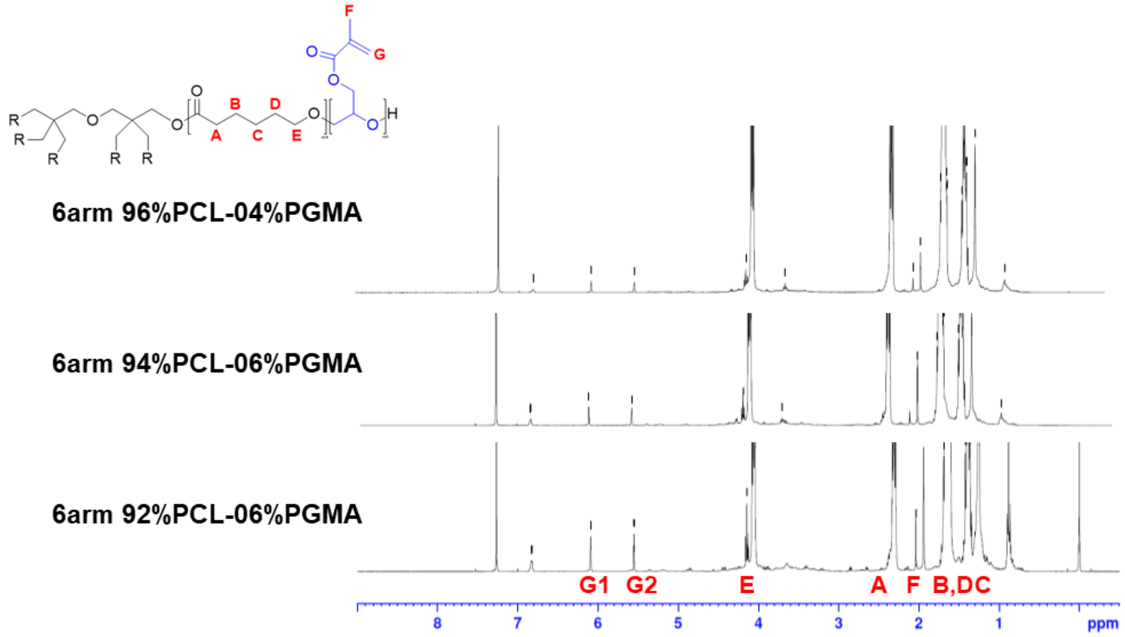

c **SMP characterization**

Samples	Before crosslinking: T_m (℃) Peak 1	Before crosslinking: T_m (℃) Peak 2	Before crosslinking: T_c (℃)	Before crosslinking: ΔH_c (J/g)	After crosslinking: T_m (℃)	After crosslinking: T_c (℃)	After crosslinking: ΔH_c (J/g)
6arm 96%PCL-04%PGMA	45.87±0.12	51.53±0.38	17.33±1.10	70.52±0.81	46.2±0.35	15.83±1.72	57.60±3.01
6arm 94%PCL-06%PGMA	44.13±0.76	50.00±0.52	17.37±1.42	65.76±4.59	38.6±2.07	1.03±8.11	43.93±4.85
6arm 92%PCL-08%PGMA	37.00±2.16	45.17±1.12	6.87±2.57	52.24±1.39	-	-	-

d **SMP degradation**

	Time (Year)	T_m	△H_m (J/g)	X_c (%)	Weight loss (%)
1 year duration	0	42.7	59.5	43.8	0
	1	42.3	52.1	38.3	21.4

Figure S3. SMP synthesis and characterization **a)** A library of 6-arm *x*% PCL-*co*-*y*% PGMA was synthesized through the ring opening polymerization of CL with GMA by controlling the molar ratio of components (% molar ratio). **b)** The structural analysis of 6-arm *x*% PCL-*co*-*y*% PGMA was confirmed by the ^1H-NMR spectrum of each polymer structure according to the molar ratio in $CDCl_3$; : δ = 6.13 [s, = CH_2, (G2)], 5.58 [s, = CH_2, (G1)], 4.10 [m, -OCH_2, (A)], 2.41 [m, -CH_2, (E)], 1.97 [s, -CH_3, (F)], and 1.74 [m, -CH_2, (B,D)], 1.45 [m, -CH_2]. **c)** Variations in the thermal properties of 6-arm *x*% PCL-*co*-*y*% PGMA were characterized by changing the molar % and crosslinking using DSC**.** d) The degradation of SMP was determined through the changes in thermal properties (DSC) and weight for one year under the accelerated aging condition following ASTM 1980.

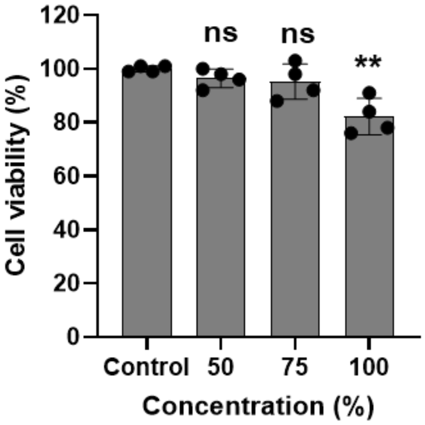

Figure S4. Cytotoxicity of SMP was determined using a CCK-8 assay after eluates of the 6-arm 94% PCL-06% PGMA in a series of dilutions (50%, 75%, and 100%) were treated with L929 cells for one day. $^{**}P < 0.005$ vs. control with no SMP treatment (N=3/ ns: not significant).

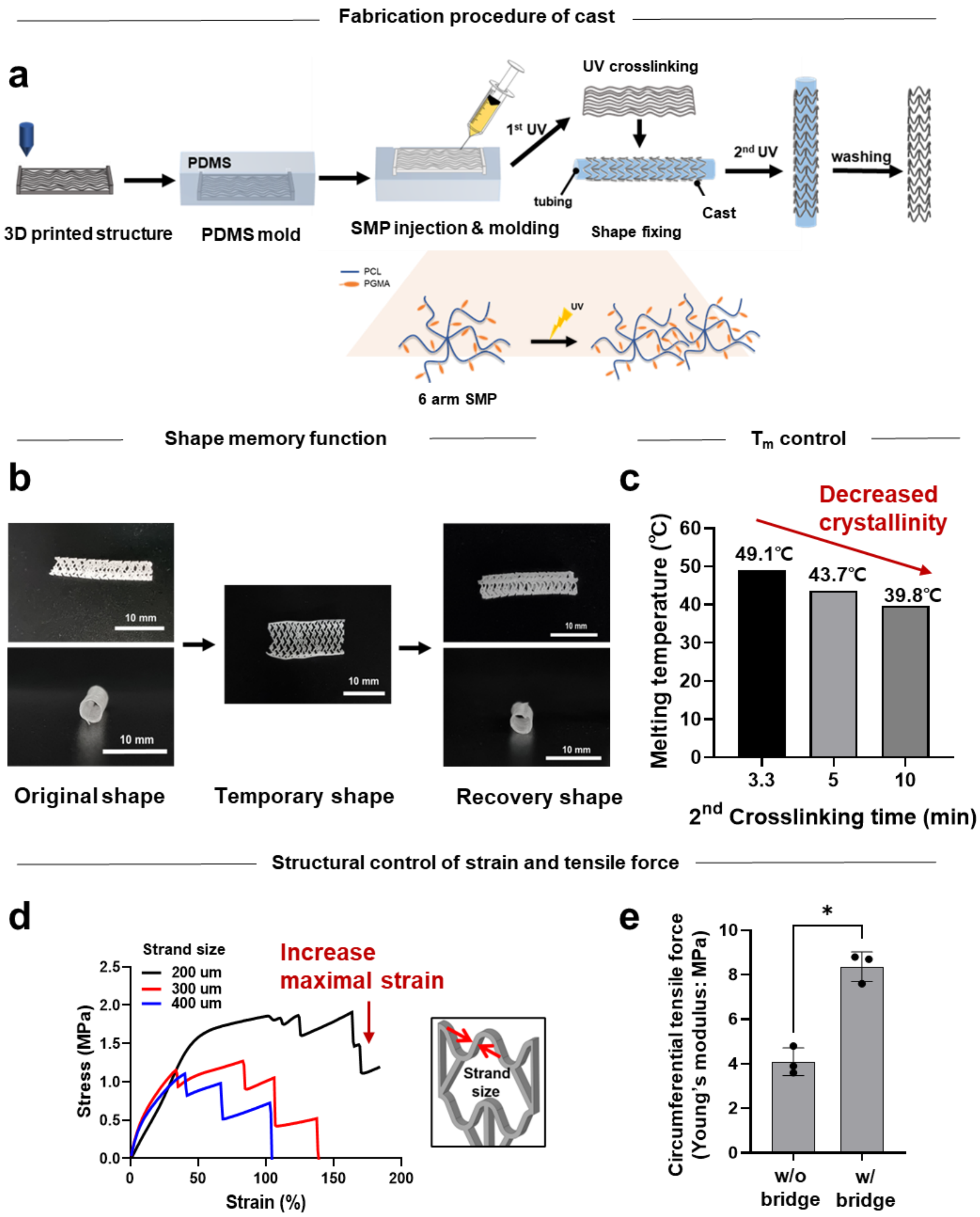

Figure S5. Vascular cast production with property tuning. **a)** The vascular cast is produced using the 6-arm 94% PCL-06% PGMA in a PDMS mold, whose structure is generated using a 3D printed model, followed by two-step crosslinking under UV. **b)** The original shape of the wrap is programmed to recover from a temporary plate shape, which facilitates deployment with covering

end-to-end anastomosis. **c)** The increased duration of the 2nd crosslinking reduces T_m to recover the shape around the body temperature owing to the reduction of the crystallinity. **d)** The strand size of the vascular cast is adjusted to 200 μm among the test sizes, as the maximal strain is increased over 150% considering the elastic synchronization with arterial contractility. **e)** The bridge increases the circumferential tensile strength to suppress vein dilation with the calculated fixity. $^{*}P < 0.01$ vs. w/o bridge (N=3).

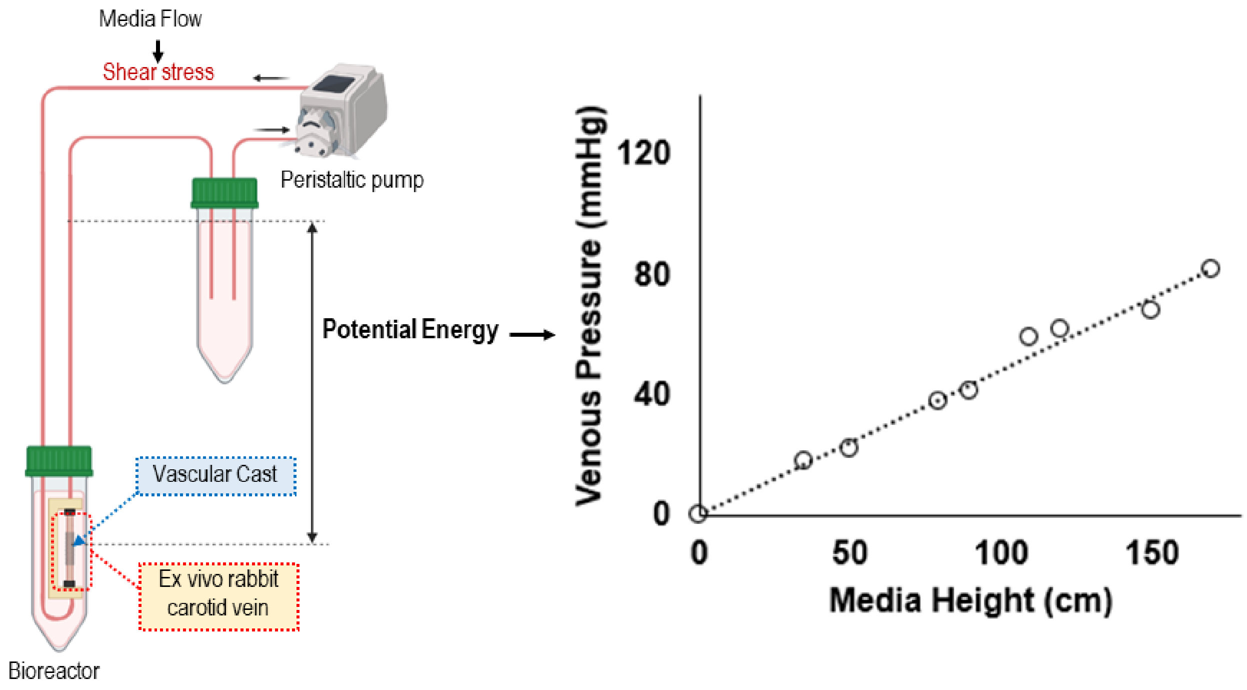

Figure S6. Ex vivo system to examine vein dilation in response to arterial hemodynamics. The system is set up to generate arterial shear stress and pressure using a peristatic pump with the tube flow control by adjusting the potential energy of media reservoir. The rabbit carotid vein, which functions as a bioreactor is loaded between the silicon tube with cast rapping in a tube. As the height of the media reservoir is elevated, the potential energy increases the venous pressure, which enables the pressure calculation.

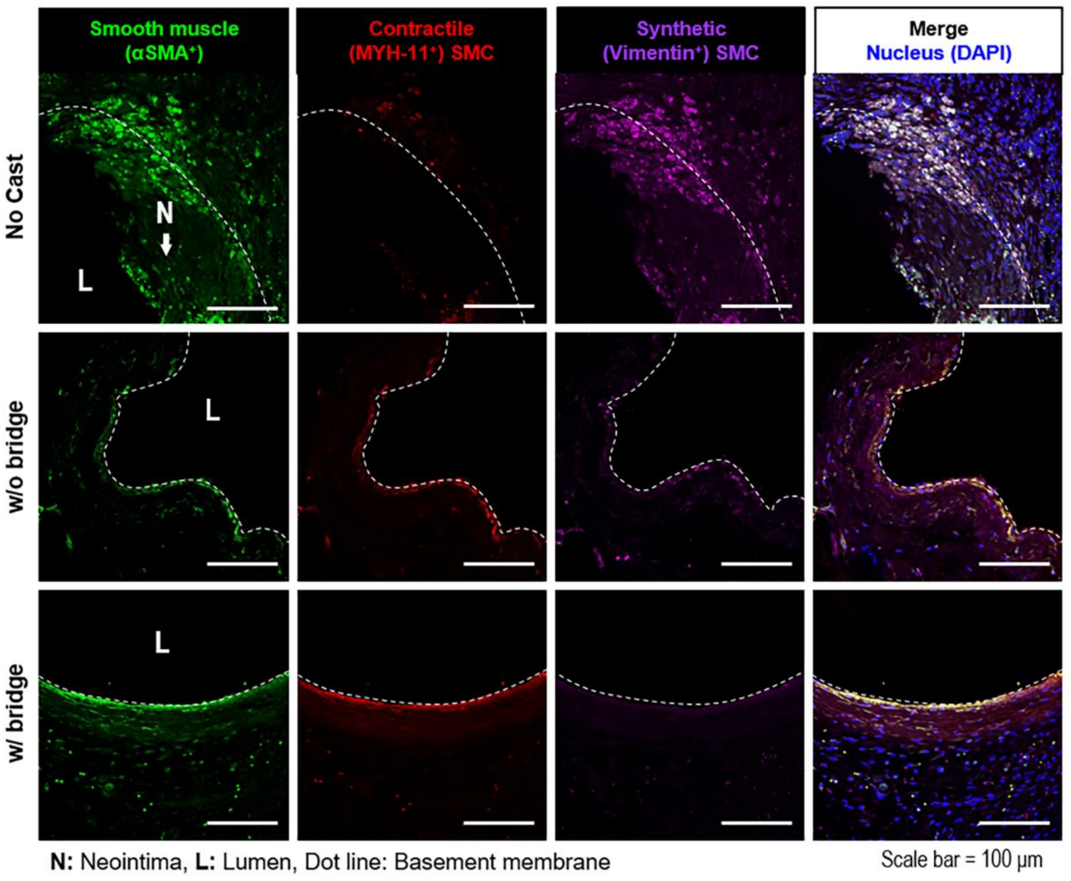

Figure S7. Phenotypic markers of smooth muscle cells in the carotid artery-vein arteriovenous fistula model of rats by immunostaining with a-SMA, MYH-11, vimentin, and nucleus.

Table S1. Definition of hemodynamic descriptors.

Wall shear stress (WSS)	$\mathrm{WSS} = \mu \frac{\partial u}{\partial y}$
Time-Averaged WSS (TAWSS)	$\mathrm{TAWSS} = \frac{1}{T}\int_0^T \lvert \mathrm{WSS} \rvert \, dt$
Oscillatory shear index (OSI)	$\mathrm{OSI} = 0.5\left[1 - \left(\frac{\left\lvert \int_0^T \mathrm{WSS}\, dt \right\rvert}{\int_0^T \lvert \mathrm{WSS} \rvert\, dt}\right)\right]$
Helicity	$H = \int_V \vec{v} \cdot \vec{w} \, dV$
Averaged Helicity intensity(h2)	$h2 = \frac{1}{TV}\int_T \int_V \vec{v} \cdot \vec{w} \, dV \, dt$
Local Normalized Helicity (LNH)	$\mathrm{LNH} = \frac{\vec{v} \cdot \vec{w}}{\lvert \vec{v} \rvert \cdot \lvert \vec{w} \rvert} = \cos\gamma$

WSS indicates the vector of the wall shear stress, μ is the viscosity of blood, u is the directional velocity of the blood flow, y is the normal direction of vessel wall, T is the period of cardiac cycle, v is the velocity vector, ω is the vorticity vector, and γ is the angle between the velocity and vorticity.

Table S2. Primers used for qRT-PCR

Species	Primer	Sequence (5'→3')
Rabbit	GAPDH	Forward: GAC CAC TTC GGC ATT GTG GA<br>Reverse: ATG CCA GTG AGT TTC CCG TT
	Ephrin B2	Forward: GTC TAG CAC AGA CGG CAA CA<br>Reverse: CGC GAT CCC CGC GAA TAA
	EphB4	Forward: ACT ACT CAG CCT TTG GCT CG<br>Reverse: CCG GAG TAG GTC TTC TGT GGA
	MMP-9	Forward: CGG AGA CGG GTA TCC TTT CG<br>Reverse: CGG CGT TTC CAA AGT ACG TG
	ENOS	Forward: AGT CCT CGA CTC CTT CGA CT<br>Reverse: GGA ACC ACT TCC ACT CCT CG
Rat	GAPDH	Forward: AAG GTC GGT GTG AAC GGA TT<br>Reverse: TGA ACT TGC CGT GGG TAG AG
	Ephrin B2	Forward: AAG TGG CCT TAT TCG CAG GG<br>Reverse: CAT TGT TGT TGC CAC CTC GC
	EphB4	Forward: CCC TCG CCA CTG CTT TAG AA<br>Reverse: CCC CCT GGA CGC TTC ATA TC
	MMP-9	Forward: GAT CCC CAG AGC GTT ACT CG<br>Reverse: GTT GTG GAA ACT CAC ACG CC
	ENOS	Forward: AAG TGG GCA GCA TCA CCT AC<br>Reverse: GCC GGC TCT GTA ACT TCC TT

References

[1] V. Crescenzi, G. Manzini, G. Calzolari, C. Borri, *European Polymer Journal* **1972**, 8, 449.
[2] Z. Li, C. Kleinstreuer, *Annals of Biomedical Engineering* **2005**, 33, 209.
[3] N. Kumar, R. Pai, M. Manjunath, A. Ganesha, S. Abdul Khader, *Journal of the Brazilian Society of Mechanical Sciences and Engineering* **2021**, 43, 1.
[4] J. Kwack, A. Masud, *Computational Mechanics* **2014**, 53, 751.
[5] D. Jodko, D. Obidowski, P. Reorowicz, K. Jóźwik, *Biocybernetics and Biomedical Engineering* **2017**, 37, 528.
[6] a)G. A. Priego-Hernández, F. Rivera-Trejo, *Atmósfera* **2016**, 29, 23; b)A. Mantha, C. Karmonik, G. Benndorf, C. Strother, R. Metcalfe, *American Journal of Neuroradiology* **2006**, 27, 1113.
[7] B. Yang, S. Kilari, A. Brahmbhatt, D. L. McCall, E. N. Torres, E. B. Leof, D. Mukhopadhyay, S. Misra, *Scientific reports* **2017**, 7, 1.
[1] Z. Li, C. Kleinstreuer, *Annals of Biomedical Engineering* **2005**, 33, 209.
[2] N. Kumar, R. Pai, M. Manjunath, A. Ganesha, S. Abdul Khader, *Journal of the Brazilian Society of Mechanical Sciences and Engineering* **2021**, 43, 1.
[3] J. Kwack, A. Masud, *Computational Mechanics* **2014**, 53, 751.
[4] D. Jodko, D. Obidowski, P. Reorowicz, K. Jóźwik, *Biocybernetics and Biomedical Engineering* **2017**, 37, 528.
[5] a)G. A. Priego-Hernández, F. Rivera-Trejo, *Atmósfera* **2016**, 29, 23; b)A. Mantha, C. Karmonik, G. Benndorf, C. Strother, R. Metcalfe, *American Journal of Neuroradiology* **2006**, 27, 1113.
[6] B. Yang, S. Kilari, A. Brahmbhatt, D. L. McCall, E. N. Torres, E. B. Leof, D. Mukhopadhyay, S. Misra, *Scientific reports* **2017**, 7, 1.
